# Compartmentalized self-replication under fast PCR cycling conditions yields *Taq* DNA polymerase mutants with increased DNA-binding affinity and blood resistance

**DOI:** 10.3389/fmicb.2014.00408

**Published:** 2014-08-14

**Authors:** Bahram Arezi, Nancy McKinney, Connie Hansen, Michelle Cayouette, Jeffrey Fox, Keith Chen, Jennifer Lapira, Sarah Hamilton, Holly Hogrefe

**Affiliations:** Agilent TechnologiesLa Jolla, CA, USA

**Keywords:** fast PCR, fast cycling, *Taq* mutants, blood resistant, inhibitor resistant

## Abstract

Faster-cycling PCR formulations, protocols, and instruments have been developed to address the need for increased throughput and shorter turn-around times for PCR-based assays. Although run times can be cut by up to 50%, shorter cycle times have been correlated with lower detection sensitivity and increased variability. To address these concerns, we applied Compartmentalized Self Replication (CSR) to evolve faster-cycling mutants of *Taq* DNA polymerase. After five rounds of selection using progressively shorter PCR extension times, individual mutations identified in the fastest-cycling clones were randomly combined using ligation-based multi-site mutagenesis. The best-performing combinatorial mutants exhibit 35- to 90-fold higher affinity (lower *K_d_*) for primed template and a moderate (2-fold) increase in extension rate compared to wild-type *Taq*. Further characterization revealed that CSR-selected mutations provide increased resistance to inhibitors, and most notably, enable direct amplification from up to 65% whole blood. We discuss the contribution of individual mutations to fast-cycling and blood-resistant phenotypes.

## Introduction

*Taq* DNA polymerase is still considered the workhorse of PCR, providing great economy and reliability in routine amplification of genomic targets up to 2 kb. Moreover, PCR detection in real-time relies on *Taq*'s intrinsic 5′-flap endonuclease activity for TaqMan probe hydrolysis and lack of proofreading activity to minimize primer/probe degradation (Holland et al., 1991). With an extension rate of 60 nt/s, wild-type *Taq* produces high amplicon yields after 30–40 cycles using 1 min anneal-extension times. Run times are 1.5–2 h on conventional Peltier-based PCR instruments and approximately 1 h using advanced qPCR instrumentation with improved thermal ramp rates (2.2–3°C/s). Demand for higher throughput and shorter turn-around-time continues to fuel interest in developing faster PCR instrumentation, along with polymerases with improved kinetic properties. In the near future, microchip-based technologies are expected to provide drastically reduced run times (<3–5 min), limited only by the kinetics of nucleotide incorporation (Hashimoto et al., 2004).

Currently, shorter qPCR run times are achieved by reducing hold times for denaturation, annealing and extension steps, and/or by using a 2-step cycling regimen with combined annealing and extension steps (at 60°C). Although run times can be cut by up to 50%, shorter cycle times with *Taq* have been correlated with lower detection sensitivity and higher failure rates when applied across a range of primer-template combinations (Hilscher et al., 2005). Results can be further improved by increasing the amount of *Taq* or other reagents, or by reducing reaction volume and using thin-walled PCR tubes to further improve heat transfer. Ultimately, however, PCR run times are limited by the kinetic properties of the PCR enzyme. In the most telling example, the processivity of a proofreading Family B DNA polymerase was directly correlated to PCR cycle times. When the processivity of *Pfu* DNA polymerase was increased by 9-fold by fusion to a small basic double-stranded DNA-binding protein (Sso7d), PCR annealing/extension times could be reduced from 2 min to 30 s for a 5 kb λ target (Wang et al., 2004). Unfortunately, this strategy could not be employed to accelerate TaqMan assays, as *Pfu* lacks 5′ endonuclease activity and Sso7d fusions to full-length *Taq* are unstable (data not shown).

To accelerate qPCR run times, we applied the Compartmentalized Self Replication (CSR) technique (Ghadessy et al., 2001) to evolve faster-cycling *Taq* mutants. CSR employs emulsion PCR to trap individual *E. coli* cells harboring mutant polymerase genes in microscopic aqueous compartments along with nucleotides and *pol* gene-specific primers. When the emulsion is cycled under selective conditions, active mutant polymerases self-replicate and are enriched, while those with insufficient activity fail to replicate and are lost from the gene pool. CSR has been used to successfully evolve *Taq* mutants with increased thermostability or heparin resistance (Ghadessy et al., 2001), and chimeric polymerases that are broadly resistant to complex environmental inhibitors or can process non-canonical primer-template duplexes and bypass lesions found in ancient DNA such as abasic sites (d'Abbadie et al., 2007). In this study, we used CSR to evolve *Taq* mutants that can self-replicate under progressively shorter extension times. As we will show, CSR selection netted *Taq* variants with a broad range of beneficial attributes, in addition to increased polymerization rate.

## Materials and methods

All molecular biology reagents were from Agilent Technologies unless otherwise noted. Oligonucleotides were purchased from Integrated DNA Technologies. Radioactive nucleotides [γ^33^P] ATP-3000 Ci/mmol-1 mCi (NEG302H001MC) and deoxythymidine-5′-triphosphate [Methyl-^3^H] tetrasodium salt-1 mCi (NET221A001MC) were purchased from Perkin Elmer.

### Random and site-directed mutagenesis

*Taq* mutants were generated by random mutagenesis of the *Thermus aquaticus pol I* gene using the GeneMorph II random mutagenesis kit and PCR primers that introduce *Xba*I and *Sal*I sites and an N-terminal His_6_ tag (F: GGCGGCTCTAGATAACGAGGGCAAAAAATGCA TCATCA TCACCATCAC, R: GCGGTGCGGAGTCGACTTACTCCTTGGCGGAGAGCCAGTC). PCRs also included 5% DSMO and increasing amounts of plasmid template (10 ng, 1 ng, 0.1 ng) to generate libraries with varying mutation rates of 4.7–6.2 per kb. After *Dpn*I treatment, PCR products were gel purified and digested with *Xba*I and *Sal*I (NEB). Purified fragments were cloned into the pASK-IBA5C expression vector (IBA) and transformed into XL10-Gold Kan cells. Site-directed mutagenesis was performed using the QuikChange Lightning or QuikChange Lightning Multi Site-Directed Mutagenesis kit.

### CSR selection

Approximately 250,000 independent clones were scraped from plates, re-suspended, and stored as glycerol stocks. LB/CAM cultures (40 ml) were freshly inoculated, grown at 30°C to OD_600_ of 0.6, and induced with 200 ng/ml anhydrotetracycline. After 3 h of growth, cells were harvested and washed in 1× *Taq* buffer. CSR was carried out essentially as described by Ghadessy et al. (2001). To select for faster-cycling mutants, extension times were successively reduced over five rounds of CSR from 2.5 min (round 1) to 15 s (round 5). PCR selection was performed on a Robocycler 96 using 2.5 min at 94°C followed by 30 cycles of 45 s at 94°C, 30 s at 60°C, and 0.25–2.5 min at 72°C.

### Protein expression

Colonies expressing mutant *Taq* polymerases were randomly picked, replicated, and then grown overnight at 30°C in 96-deep well plates (VWR) containing 750 μl LB/CAM. Overnight cultures (30 μl) were inoculated into fresh media, induced with anhydrotetracycline at OD_600nm_ of 0.3–0.5, and grown overnight with shaking at 30°C. Cells were collected and used to prepare lysates for direct PCR screening or for affinity protein purification (see below). Cell pellets were re-suspended in 50 μl Tris pH 8 containing 4 mg/ml lysozyme, and incubated at 37°C for 10 min to disrupt cell walls and at 75°C for 15 min to inactivate *E. coli* protein. Lysates were clarified by centrifugation for 15–30 min at 4000 RPM through a 96-well filter plate (Millipore, Multiscreen HTS, HV).

### Purification of his-tagged *taq* mutants

Cell pellets were re-suspended in 90 μl of buffer prepared by adding one cOmplete EDTA-free Protease Inhibitor tablet (Roche) to 50 ml of 50 mM Tris pH 8, 0.5 M NaCl, 5 mM imidazole. An aliquot (60 μl) of 1× FastBreak Cell Lysis Reagent (Promega) was added and the lysate was incubated at 37°C for 15 min and at 70°C for 15 min, before centrifugation through a Millipore 96-well filter plate. Clarified lysates were combined with 60 μl Ni-NTA agarose (Qiagen) and incubated with shaking at room temperature for 2 h. After collecting the agarose resins using a fresh filter plate, resins were washed two times with 200 μl wash buffer (50 mM Tris pH8, 0.5 M NaCl, 20 mM imidazole) and eluted with 80 μl of 50 mM Tris pH8, 0.5 M NaCl, 200 mM imidazole.

### Fast PCR screening

*Taq* mutants were screened by amplifying a 549 bp GAPDH target on the Mx3005 qPCR system using fast-cycling conditions consisting of 1 min at 95°C followed by 50 cycles of 2 s at 99°C, 7 s at 59°C. PCRs (25 μl) contained 1× *Taq* buffer (15 mM Tris pH 8.0, 50 mM KCl, 2.5 mM MgCl_2_, 0.01% Tween-20), 0.8 mM dNTPs, GAPDH primers (5′-ATCTTGAGGCTGTTGTCATAC; 5′-CAGGAAACAGCTATGACCATG), 10^5^ copies plasmid DNA, 0.8× Eva Green, and either 2 μl clarified lysate (neat or diluted 1:5) or 10–50 ng of purified His-tagged *Taq*. Primary hits displayed earlier C_q_s compared to wild-type *Taq* controls processed in the same way on the same plates.

### Column purification of non-tagged *taq* mutants

Mutant *pol* genes were subcloned into pET11 (with no His tag) and expressed in *E. coli* strain BL21-DE3-RIPL. One liter cultures were grown at 30°C in LB medium with 125 μg/ml ampicillin and 30 μg/ml chloramphenicol, and induced at OD_600nm_ of 0.6 with 1 mM IPTG for 4–5 h. Cell pellets were recovered by centrifugation and stored at −20°C. For purification, pellets were suspended in Buffer A (50 mM Tris-Cl pH 8.2, 1 mM EDTA, 10 mM 2-mercaptoethanol) plus cOmplete Protease Inhibitor (Roche). Cell suspensions were disrupted by sonication, brought to 0.2 M with solid (NH_4_)_2_SO_4_, heated in a water bath at 80°C for 15 min, and then cooled on ice. Polyethyleneimine was added to 0.2% (w/v), and after mixing thoroughly, insoluble material was removed by centrifugation. The supernatant was loaded on an SP Sepharose FastFlow (GE Healthcare) column equilibrated and run with Buffer B (50 mM Tris-Cl pH8, 1 mM EDTA, 0.2 M (NH_4_)_2_SO_4_, 10 mM 2-mercaptoethanol, 5% glycerol). Flow-through fractions were pooled and dialyzed against 15 volumes (with two changes) of Buffer C (50 mM Tris-Cl pH8.3, 1 mM EDTA, 10 mM 2-mercaptoethanol, 5% glycerol), and then loaded on Q Sepharose HP (GE Healthcare) equilibrated with Buffer C. The column was eluted with a 12.5 column-volume gradient to Buffer C containing 400 mM KCl. *Taq*-containing fractions (as judged by SDS-PAGE) were pooled, diluted with 2.5 volumes of Buffer D (50 mM Tris-Cl pH 7.5, 1 mM EDTA, 10 mM 2-mercaptoethanol, 5% glycerol, 125 mM KCl) and then loaded on Heparin Sepharose HP (GE Healthcare). The column was eluted with a 25 column-volume gradient to Buffer D with 650 mM KCl. Substantially-pure *Taq*-containing fractions were pooled, dialyzed into storage buffer (20 mM Tris-Cl pH 8, 0.1 mM EDTA, 1 mM DTT, 100 mM KCl, 50% glycerol), and stored at −20°C. Protein was quantified using the Coomassie Plus protein assay (Thermo Fisher Scientific).

### Real-time PCR assays

SYBR Green qPCR reactions (20 μl) consisted of 10–20 ng wild-type or mutant *Taq*, 0.5–50 ng human genomic DNA, 200 μM each dNTP, 600 nM total primers, 1× *Taq* buffer (adjusted to 95 mM KCl for all PCRs and biochemical assays employing *Taq* mutants), and 0.24× SYBR Green. Primer sequences were as follows: ABC (F: 5′-CCAAACCCTGGATCACGTGTT-3′; R: 5′-CCTCCGCGTCTCGTAGTTCT-3′), COMTE2 (F: 5′-GAGATCAACCCCGACTG-3′; R: 5′-GGCCCTTTTTCCAG-3′), Quantos (F: 5′-TATAAGAAACTACTAAGCACCCAAAGG-3′; R: 5′-AAGAAAGGAGTCTAAGTGACTCAACAG-3′; Aldolase (F: 5′-AGCCTAGCTCCAGTGCTTCTAGTA-3′; R: 5′-CTTTGGATGAGGAGCCGATATTG-3′), Numb (F: 5′-GAGGTTCCTACAGGCACCTGCCCAG-3′; R: 5′-CAAAATCACCCCTCACAGTACTCTG-3′). TaqMan qPCR reactions consisted of 10 ng wild-type or mutant *Taq*, human genomic DNA, 0.8 mM dNTPs, 1× *Taq* buffer (adjusted to 95 mM KCl for *Taq* mutants), and 1× β-actin primer/probe (171 bp) from Life Technologies' Assays-On-Demand. qPCR reactions were run on the StepOnePlus (Life Technologies) or CFX96 (BioRad) instrument using cycling parameters indicated in the Figure legends.

### Primer extension assays

Extension rate and processivity were measured at 70°C using M13mp18 template DNA (NEB), pre-annealed at a 1.3–1.5:1 molar ratio to a 5′ ^33^P-labeled primer with the sequence 5′-GGTTTTCCCAGTCACGACGTTGTAAAACGACGGCCAGTGC-3′. Extension reactions (80 μl) consisting of 1.75 pmol primed M13, 200 μM each dNTP, and 1× *Taq* buffer were brought to 70°C prior to adding 0.55 pmol *Taq* enzyme. Aliquots (8 μl) were removed at 15, 30, 45, 60, 90, and 120 s and quenched with 50 mM EDTA. Extension products were denatured at 80°C for 3 min and analyzed on 1% alkaline-agarose gels to determine mean fragment length. Extension rates were calculated as number of nucleotides incorporated divided by incubation time. Processivity assays were conducted similarly using 1.1 pmol primed M13 and limiting amounts of *Taq* (template/enzyme molar ratios of 37:1, 370:1, 3700:1). Aliquots removed at various time points (15, 30, and 60 s) were quenched in gel loading buffer, and analyzed on 6% TBE-Urea gels (Life Technologies). Median processivity was determined from reactions producing the same product length over several time points or enzyme amounts.

### Nucleotide incorporation assays

Polymerase cocktails (10 μl) contained 200 μM dATP, dGTP and dCTP, 100 μM TTP (^3^H-TTP), 1× *Taq* buffer (adjusted to 95 mM KCl for *Taq* mutants), and fixed or varying concentrations of primed (non-radiolabeled) M13 DNA for half-life (T_1/2_) or *K_cat_* measurements, respectively. Steady-state kinetic parameters were determined using 0.005 pmol *Taq* and 0.5–100 nM primed M13. After 3 min at 60°C, incorporation reactions were quenched with ice-cold 0.1 M EDTA and 5 μl aliquots spotted on DE-81 filters. After washing 5 times with 2× SSC, incorporated radioactivity was measured by scintillation counting. *K_m_* and *V_max_* values were determined from Lineweaver-Burk plots, and *K_cat_* was calculated as maximum number of nucleotides incorporated per *Taq* molecule per second (*V_max_*/[*Taq*]). To determine T_1/2_(95°C), *Taq* was pre-heated in the absence or presence of genomic DNA before residual DNA polymerase activity was assayed as described above. Mixtures (10 μl) consisting of 0.02 pmol *Taq*, 1× *Taq* buffer, and 0 or 10 ng human genomic DNA were overlaid with mineral oil and incubated at 95°C for 5–180 min. At various time points, aliquots (2 μl) were transferred to polymerase assay cocktail (10 μl) containing 0.05 pmol primed M13. Polymerase activity (CPM) was plotted against pre-incubation time at 95°C.

### K_*d*_ assay

Dissociation constant *K_d_* (DNA) was measured using a gel mobility-shift assay that employs a 5′ ^33^P-labeled hairpin template (5′-CTCCAGACACGACGCAGTTGCCCGATGGTCGACGTTCGCGAAAGCGAACGTCGACCATCGGGCAACT-3′) blocked at the 3′ end with dideoxy TMP. The radiolabeled hairpin (0.25 nM) was incubated with varying concentrations of wild-type or mutant *Taq* DNA polymerases (0.5–1000 nM; bracketing the predicted *K_d_*) in 1× *Taq* buffer at 37°C for 30 min. DNA-protein complexes were run on 10% non-denaturing TBE gels (Life Technologies) in 0.5× TBE buffer. Gels were dried down and exposed to film. The fraction of DNA bound was quantified by densitometry using AlphaView SA software (Alpha Innotech), and then plotted against enzyme concentration to determine *K_d_* values by interpolation.

### PCR/qPCR inhibition assays

Xylan, humic acid, CTAB, and dextran sulfate were purchased from Sigma. Blood was collected from healthy human volunteers using BD Vacutainer tubes with EDTA or heparin. Inhibitor-resistance screens (“spike-in” assays) were conducted by adding varying concentrations of inhibitors to qPCRs (20 μl) containing 20 ng wild-type or mutant *Taq* DNA polymerase, 200 μM each dNTP, 1× *Taq* buffer, 0.24× SYBR Green, 10^5^ copies plasmid DNA, and 600 nM GAPDH primers (5′-ATCTTGAGGCTGTTGTCATAC; 5′-CAGGAAACAGCTATGACCATG) designed to amplify 549 bp GAPDH target on the Mx3005 qPCR system using 2 min initial denaturation at 95°C followed by 40 cycles of 12 s at 95°C and 60 s at 60°C.

Endpoint PCRs directly from blood (endogenous targets) consisted of (50 μl) 50 ng DNA polymerase, 15 mM Tris pH 8.8, 50 mM (wild-type *Taq*) or 95 mM (mutant *Taq*) KCl 2.5 mM MgCl_2_, 0.02% Tween-20, 200 μM each dNTP, and 2% DMSO. A 322 bp IGF target was amplified directly from blood using 400 nM each primer (IGF322-F 5′-ATGGAGGGACCAATAGTAGGGAA; IGF322-R 5′-AGTACCACGTACAGGCTTTGCAT), and the following cycling parameters: 5 min at 90°C followed by 30 cycles of 30 s at 95°C, 30 s at 60°C, 1 min at 72°C. Water (25 μl) was added to PCRs containing blood before centrifugation to pellet debris. A portion (12 μl) of each sample was run on a 4% Nusieve® (3:1) TBE agarose gel (Lonza). Comparisons employing commercial enzymes were conducted using the 232 bp Quantos (see above) assay system and each manufacturer's recommended reaction buffer and cycling conditions.

## Results

### *Taq* mutagenesis and screening

We used the CSR technique to evolve mutants of *Taq* DNA polymerase that self-replicate using abbreviated extension times. Faster-replicating mutant polymerases are expected to provide robust performance with “fast” PCR instruments and cycling conditions. Moreover, identifying mutations that allow shorter cycle times may provide insight on kinetic factors that limit PCR performance of wild-type *Taq*.

*Taq* mutant libraries were subject to CSR selection using progressively shorter extension times, ranging from 1 min down to 6 s per kb of *taq pol I*. After rounds 2, 4, and 5, crude protein preparations were prepared and tested in amplifications employing 2-step cycling with 2 s denaturation and 7 s annealing-extension times. To account for differences in protein expression, lysates were prepared and assayed at least 2–3 times before affinity-purifying the top-performing *Taq* mutants. From a screen of several hundred clones, we recovered 24 His-tagged *Taq* mutants that consistently produce earlier C_q_ values compared to wild-type *Taq* in real-time PCR under fast cycling conditions. DNA sequencing identified 8 mutations that appear in 2 or more independent clones, as follows (in order of frequency): E507K (11); G59W (9); L245M (5); V155I, L375V, F749I (4); K508R, E734G (2).

To identify the most effective mutation or combination of mutations, we performed multi-site mutagenesis with an equimolar mixture of 8 mutant primers to create all possible (single, double, triple, etc.) combinations of G59W, V155I, L245M, L375V, E507K, K508R, E734G, and F749I mutations. The combinatorial library was enriched by 1 round of CSR selection using 15 s (6 s per kb) extension times, and random clones were screened as described above with a modified *Taq* PCR reaction buffer. We increased KCl concentration from 50 to 95 mM to eliminate non-specific amplification products that were generated by the majority of fast-cycling mutants (<30% of total amplified product as judged by agarose gels or qPCR melt curves; data not shown). Higher KCl concentrations (>50 mM) inhibit wild-type *Taq* DNA polymerase to the extent where no PCR products are generated at 95 mM KCl. The most active *Taq* mutants (1C2, 2C2, 3B, 42; see Table 1) were sub-cloned to remove the His-tag, purified using a standard wild-type *Taq* protocol, and characterized further by PCR.

**Table 1:** ***Taq* mutations**.

**Enzyme**	**G59W**	**V155I**	**L245M**	**L375V**	**E507K**	**K508R**	**E734G**	**F749I**
*Taq (wt)*	−	−	−	−	−	−	−	−
*Taq* 42	+	−	+	+	+	+	+	+
*Taq* 3B	−	+	+	−	+	−	−	+
*Taq* 2C2	+	+	+	+	+	−	+	+
*Taq* 1C2	+	+	+	−	+	−	−	+

**Figure 1:**
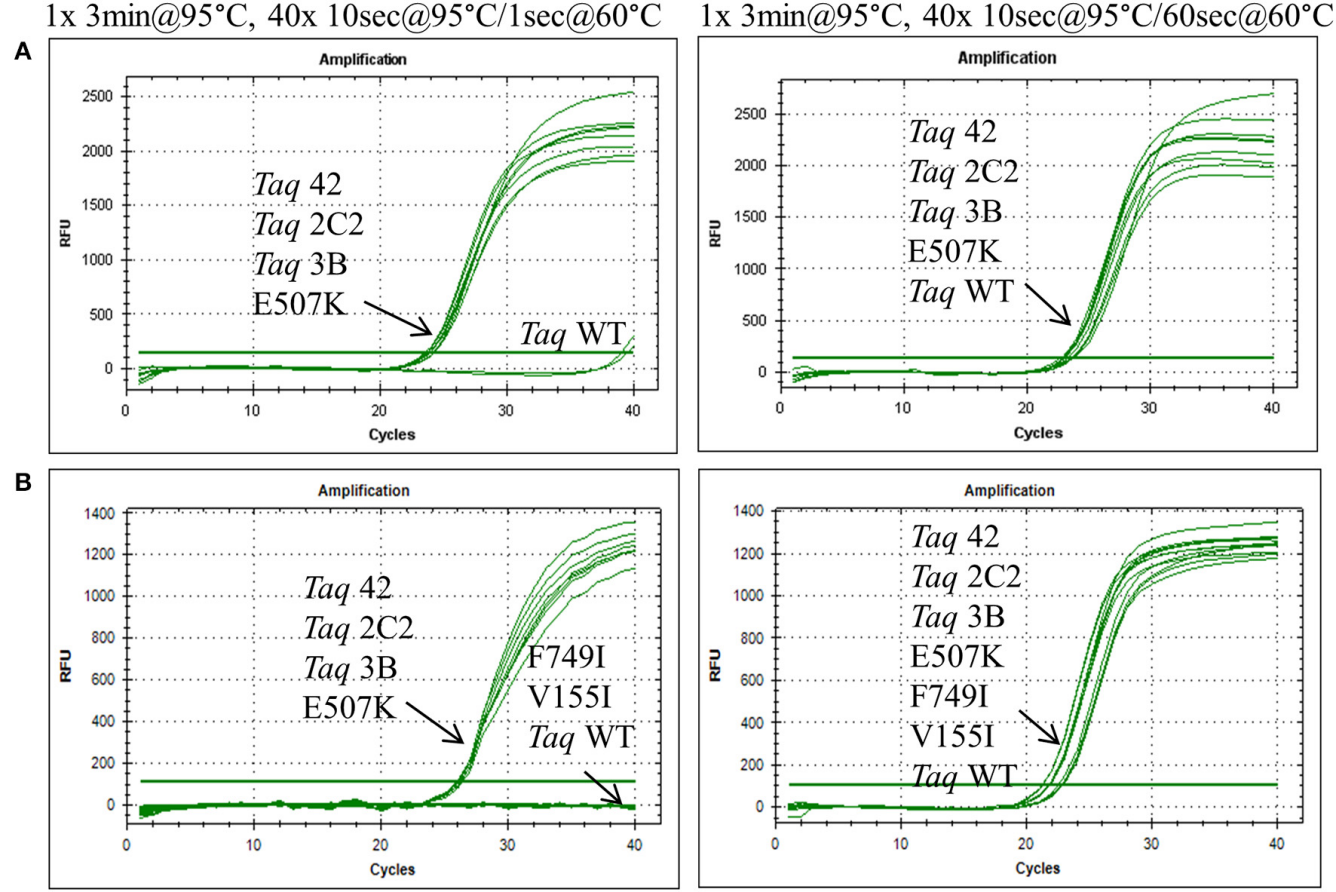
**Fast cycling conferred by E507K mutation.** Genomic DNA targets were amplified as described in Methods using 10 ng human gDNA and 20 ng of purified wild-type or mutant *Taq* DNA polymerase. Reactions were cycled on the CFX96 instrument (BioRad) using cycling conditions consisting of 3 min at 95°C followed by 40 cycles of (left panels) 10 s at 95°C, 1 s at 60°C; or (right panels) 10 s at 95°C, 60 s at 60°C. Genomic DNA targets are as follows: **(A)** 286 bp Aldolase; and **(B)** 232 bp Quantos.

**Figure 2:**
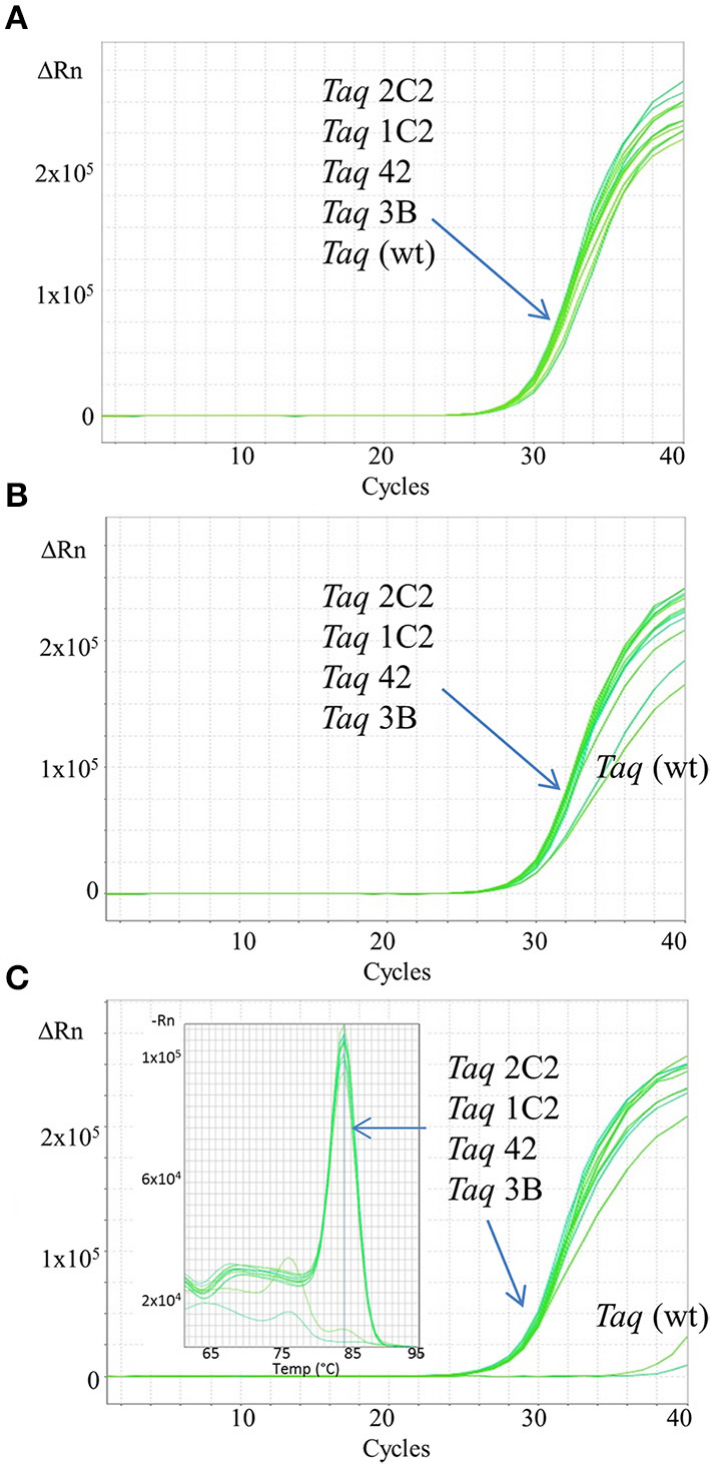
**Fast qPCR assays employing SYBR Green.** Genomic DNA targets were amplified as described in Methods using 0.5 ng human gDNA and 10 ng of purified wild-type or mutant *Taq* DNA polymerase. Reactions were cycled on the StepOnePlus instrument (Life Technologies) using cycling conditions consisting of 3 min at 95°C followed by 40 cycles of 3 s at 95°C, 10 s at 60°C. Genomic DNA targets are as follows: **(A)** 91 bp ABC, **(B)** 109 bp COMTE2, **(C)** 305 bp Numb. Sixty second extension time is required for *Taq* wild-type to efficiently amplify the 305 bp target (data not shown).

**Figure 3:**
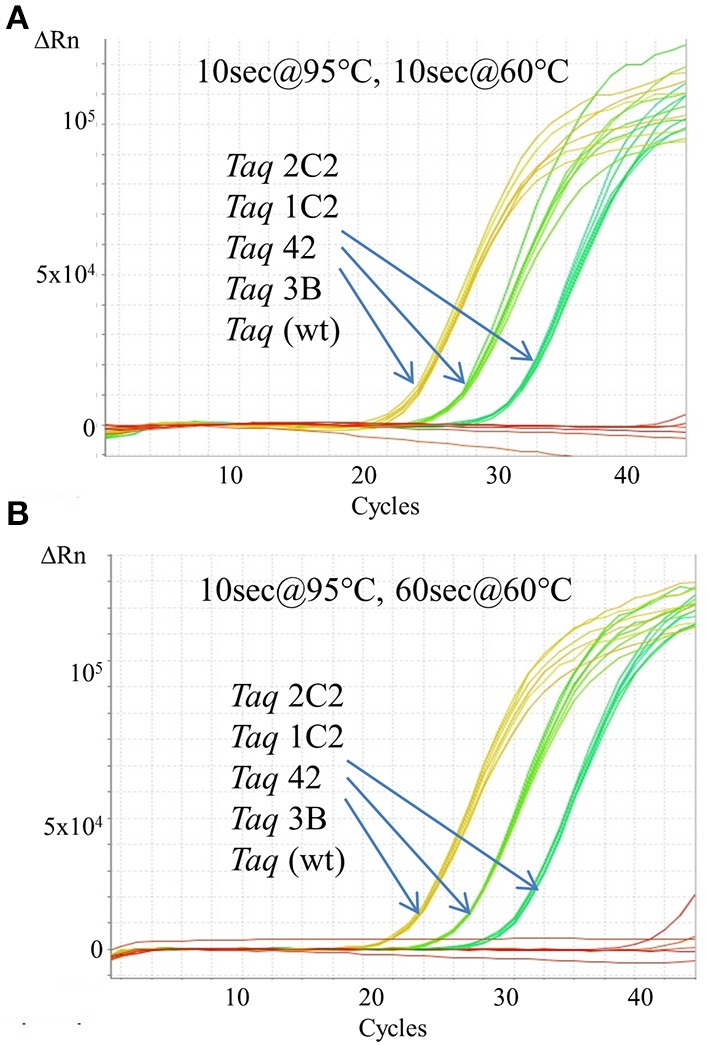
**Fast cycling with TaqMan detection.** An Assays-on-Demand assay (Life Technologies; 171 bp β-actin target) was performed using 50, 5, 0.5 ng human genomic DNA and 10 ng of purified wild-type or mutant *Taq* DNA polymerase. Reactions were cycled on the StepOnePlus instrument using cycling conditions consisting of 2 min at 95°C followed by 40 cycles of: **(A)** 10 s at 95°C, 60 s at 60°C; or **(B)** 10 s at 95°C, 10 s at 60°C.

In endpoint PCRs employing longer (>1 kb) genomic targets, *Taq* 1C2, 2C2, 3B, and 42 efficiently amplified targets using 15–30 s/kb extension times whereas *Taq* required 1 min/kb to generate similar yields (data not shown). *Taq* 1C2, 2C2, 3B, and 42 were also tested with shorter targets to assess the benefits of fast cycling in quantitative PCR (qPCR) assays that traditionally employ targets of <300 bp (SYBR Green detection) or <200 bp (probe detection) to achieve amplification efficiencies as close to 100% as possible. In qPCRs employing SYBR Green, *Taq* readily amplifies genomic DNA targets up to 300 bp using standard anneal-extension times of 1 min at 60°C (Figure 1). However, with 10 s anneal-extension times, *Taq* produced slightly lower ΔRn values in the 109 bp COMTE assay and completely failed to amplify a 305 bp NUMB target (Figures 2B,C). In contrast, *Taq* 1C2, 2C2, 3B, and 42 amplified the entire series (91–305 bp) of genomic DNA targets using abbreviated cycle times (10 s, Figure 2; 1 s, Figure 1), and no significant differences were observed among the mutants with respect to C_q_s, ΔRn values, and amplification efficiencies. Compared to SYBR Green assays, abbreviated cycle times appear to have less of an impact in TaqMan assays designed according to standard primer-probe design rules (<200 bp targets). In the example shown in Figure 3, *Taq* 1C2, 2C2, 3B, and 42 produce equivalent results to wild-type *Taq* using 10 s anneal-extension times, thereby confirming that probe-hydrolysis (5′-structure-specific endonuclease) activity is not affected by the fast-cycling mutations.

### Mutations conferring the fast-cycling phenotype

**Figure 4:**
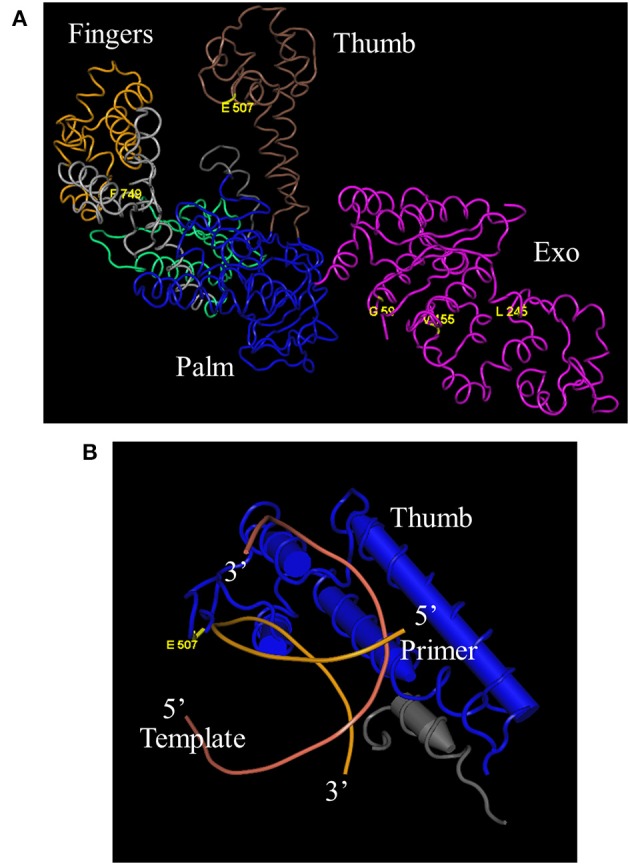
**Location of E507K and blood-resistant mutations in *Taq* crystal structure.** “Fitness mutations” G59W, V155I, L245M, and F749I reside in the 5′–3′ exonuclease and Fingers domains, respectively, while fast-cycling mutation E507K is in the thumb domain **(A)** (Kim et al., 1995). E507 resides in close proximity to the primer portion of the primer-template duplex **(B)** (Li et al., 1998).

DNA sequencing revealed that 3 of the original 8 mutations-L245M, E507K, and F749I- are absolutely conserved in *Taq* 1C2, 2C2, 3B, and 42, indicating that one or more are essential for fast-cycling under CSR selection conditions (Table 1). While not critical, other mutations emerging from CSR selection may further enhance overall fitness of the *Taq* 245M/507K/749I mutants. For example, G59W (missing in *Taq* 3B) and/or V155I (missing in *Taq* 42) appear in majority (3 out of 4) of the top-performers, suggesting a role for these N-terminal domain mutations in enhancing robustness. The L375V, K508R, and E734G mutations appear less frequently, and may be of limited importance to survival during CSR selection. Figure 4A shows the location of G59W, V155I, and L245M in the N-terminal 5′–3′ endonuclease domain, and the close proximity of E507K and F749I in the thumb and fingers domains, respectively.

To further elucidate the contribution of individual mutations to the fast-cycling phenotype, we constructed and purified individual mutants *Taq* G59W, *Taq* V155I, *Taq* E507K, and *Taq* F749I. When equivalent amounts of enzyme were compared in qPCRs, only *Taq* E507K amplified 286 and 232 bp genomic DNA targets with short (1 s) anneal-extension times (Figure 1 and data not shown). Moreover, *Taq* E507K produced equivalent C_q_ values to *Taq* 42, 2C2, and 3B, indicating that the E507K mutation is solely responsible for the fast-cycling phenotype. With standard cycle times (60 s anneal-extension), amplification profiles are comparable across all *Taq* enzymes, confirming that single-site mutants *Taq* G59W, *Taq* V155I, and *Taq* F749I retain wild-type levels of polymerase activity.

### Kinetic parameters of *taq* mutants

Kinetic parameters were investigated at each enzyme's KCl optimum (50 or 95 mM for *Taq* or *Taq* mutants, respectively) to determine how E507K confers the fast-cycling phenotype. In radio-labeled primer extension assays, *Taq* E507K and *Taq* 42 exhibit somewhat higher (1.7-fold) polymerization rates compared to wild-type *Taq* (85 vs. 50 nt s^−1^; Table 2), but no change in processivity (20 bases; Table 2). Measurements of steady-state kinetic parameters also show a moderate (2.2-fold) increase in *K_cat_* values (2.5 ± 0.03 s^−1^ for *Taq* 42; 1.1 ± 0.04 s^−1^ for *Taq*; data not shown). The most compelling difference between wild-type and E507K mutants was observed in gel-based *K_d_* assays employing a hairpin oligonucleotide template. As shown in Table 2, dissociation constants for *Taq* 42, 1C2, and E507K mutants (2.75, 1.9, and 1.1 nM, respectively) were approximately 35–90-fold lower compared to those of *Taq* and *Taq* G59W (102 and 91 nM, respectively). Moreover, *K_d_* measurements were comparable for assays run in the absence or presence of dNTPs (data not shown). In sum, these results indicate that the E507K mutation supports faster PCR cycling conditions by increasing binding affinity of *Taq* for primed DNA templates irrespective of nucleotide binding.

**Table 2:** **Kinetic parameters of wild-type and mutant *Taq* polymerases**.

**Enzyme**	**Extension rate (nt/s)**	**Processivity (nt)**	***K_d_* [DNA] (nM)**
*Taq (wt)*	50	20	102
*Taq* 42	85	20	2.75
*Taq* 1C2	ND	ND	1.9
E507K	85	20	1.1
G59W	ND	ND	91.6

*ND, Not Determined*.

### Mutations improving polymerase fitness

CSR has been shown to exert strong selective pressure on polymerase fitness, enriching for variants that self-replicate with sufficient accuracy and efficiency to remain in the gene pool (Ghadessy et al., 2001). In some cases, selected traits evolved in parallel with increased inhibitor resistance, presumably because survival requires self-replication in the presence of CSR emulsifiers/stabilizers and bacterial debris (Baar et al., 2011). In our study, several mutations including G59W, V155I, L245M, and F749I emerged during selection of the fast-cycling phenotype, prompting speculation that one or more of these ancillary mutations may enhance overall fitness of *Taq* DNA polymerase. To address this possibility, we further characterized the stability and inhibitor-resistance of a subset of *Taq* multi-site mutants.

Thermostability was investigated by determining half-life (T_1/2_) at 95°C, in the absence and presence of DNA template. Compared to wild-type, *Taq* 42 exhibits a slightly higher T_1/2_ in the presence of DNA (71 ± 4.6 vs. 60 ± 1.7 min), but no significant difference in the absence of DNA (60.5 ± 2.3 vs. 62 ± 2 min; data not shown). These results are consistent with increased thermal resistance conferred by tighter binding (of E507K mutants) to DNA, but also imply that none of the other mutations in *Taq* 42 (G59W, V155I, L245M, L375V, K508R, E734G, F749I) enhance intrinsic thermal resistance. Next, inhibitor resistance of *Taq* 2C2 (all mutations except K508R) was investigated by amplifying an exogenous target in the presence of varying amounts of known *Taq* inhibitors, including plant-associated substances (xylan, dextran sulfate, CTAB), soil inhibitors (humic acid), whole blood treated with various anti-coagulants, and other PCR inhibitors (NaCl, SYBR Green) (Demeke and Adams, 1992; Watson and Blackwell, 2000; Kermekchiev et al., 2009). In these studies, inhibitor resistance was determined relative to wild-type *Taq* using standard PCR cycling times. Compared to wild-type *Taq, Taq* 2C2 shows significantly (≥4-fold) higher tolerance to dextran sulfate (50-fold; data not shown), NaCl (4-fold; Table 3), and whole blood (see below).

**Table 3:** Resistance of *Taq* mutants to blood and NaCl^*^.

**Enzyme**	**EDTA blood (%)**	**Heparinized blood (%)**	**NaCl (mM)**
*Taq* (wt)	<1	<1	25
*Taq* 42	60	10	100
*Taq* 3B	50	10	100
*Taq* 2C2	65	25	100
*Taq* 1C2	60	30	100
E507K	45	2	100
F749I	15	2	25
V155I	15	<2	25
G59W	15	2	10

* *The values represent the highest inhibitor concentration beyond which PCR amplification is inhibited*.

**Figure 5:**
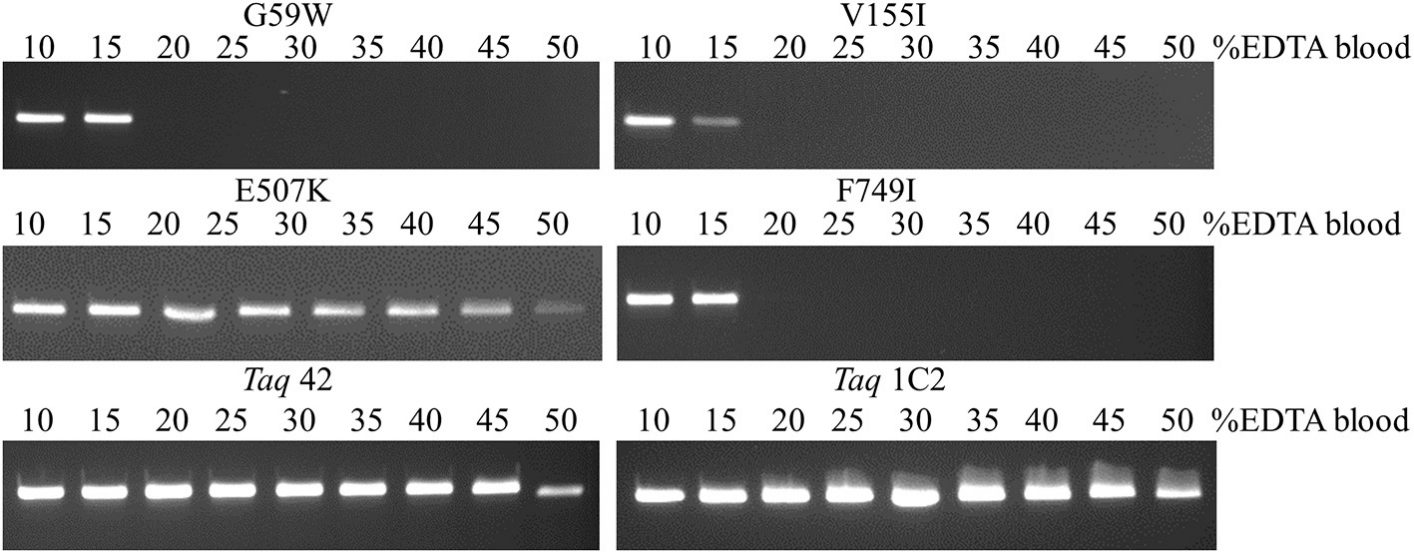
**Amplification directly from blood.** A 322 bp IGF target was amplified from 5 to 25 μl EDTA-blood using 50 ng of each mutant *Taq* DNA polymerase. Reactions were cycled on the SureCycler 8800 using the following parameters: 5 min at 90°C, followed by 30 cycles of 30 s at 95°C, 30 s at 60°C, 60 s at 72°C. Wild-type *Taq* can amplify the 322 bp from human genomic DNA in the absence of blood, but not in the presence of 1% blood (data not shown), even though the amount of EDTA introduced with 0.5 μl EDTA-blood (0.089 mM EDTA) is well below inhibitory levels.

Additional testing with single- and multi-site mutants revealed that E507K confers increased tolerance to NaCl (up to 100 mM) in addition to higher affinity for primed-template. In contrast, mutations at other positions result in equivalent (V155I, F749) or reduced (*Taq* G59W; by 2.5-fold) tolerance to NaCl compared to wild-type *Taq*. Next, resistance to blood-associated inhibitors was investigated in more depth by amplifying endogenous targets directly from blood (EDTA tubes). As shown in Figure 5, the E507K mutation also provides increased tolerance to blood, allowing amplification of a 322 bp IGF target from up to 45% EDTA-blood. Resistance to blood may be partly explained by higher salt tolerance, as the amount of NaCl introduced with 22.5 μl blood is within the range (up to 100 mM; Table 3) tolerated by *Taq* E507 (67.5 mM NaCl final). Interestingly, individual mutations at G59W, V155I, and F749I also confer significant resistance to EDTA-blood, albeit less than E507K (15% compared to 45%), perhaps reflecting intrinsic sensitivity to NaCl (tolerate <10–25 mM, while 15% blood introduces 22.5 mM NaCl). In contrast, wild-type *Taq* fails to amplify the 322 bp endogenous target from as little as 1% EDTA-blood (Table 3), consistent with previous reports (Al-Soud and Radstrom, 1998; Kermekchiev et al., 2009). In total, these results indicate that G59W, V155I, and F749I mutations confer significant (>10–15-fold) resistance to blood-associated inhibitors through a mechanism that is distinct from E507K (unrelated to increased binding affinity and salt resistance). Moreover, when the majority of CSR-selected mutations are added to E507K, resistance increases from 45% (*Taq* E507K) to between 50% (*Taq* 3B; 3 additional mutations) and 60–65% (*Taq* 1C2, 2C2, 42; 4–6 additional mutations).

**Figure 6:**
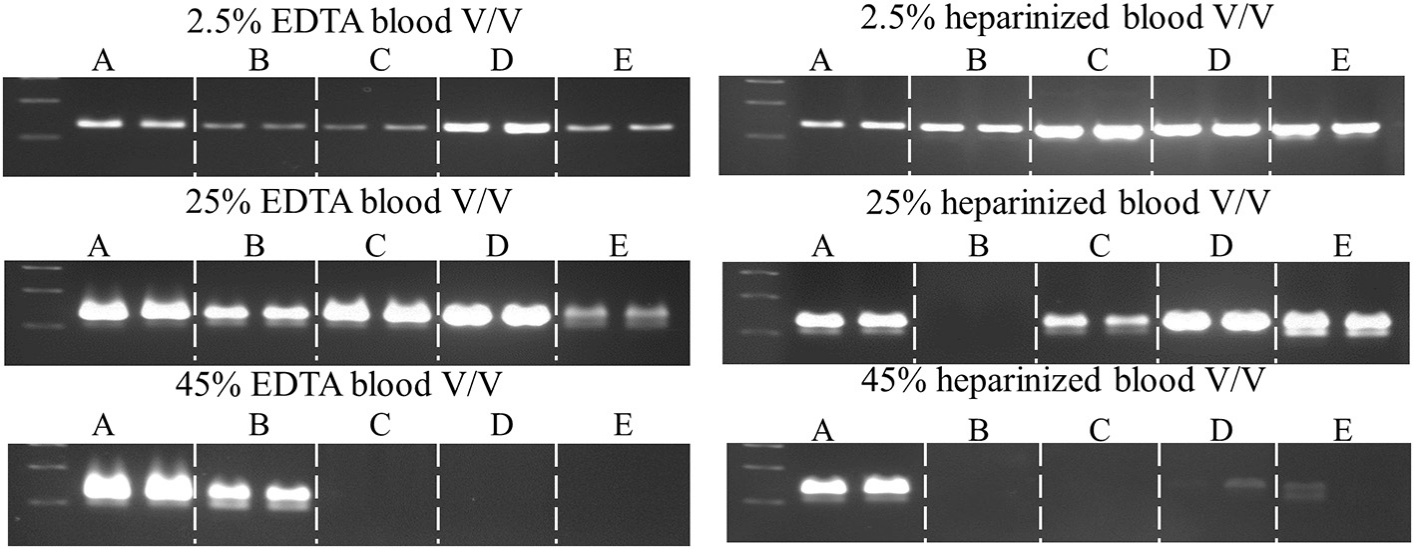
**Comparison of commercial enzymes for blood PCR.** A 232 bp single-copy target was amplified (50 μl) from the indicated volumes of blood using SureDirect PCR mix **(A)** or another commercially-available PCR blood kits **(B–E)**. All reactions contained 0.4 μM each Quantos primer and were cycled on a SureCycler 8800 instrument, according to each manufacturer's recommendations. PCRs performed with SureDirect PCR master mix were cycled as follows: 5 min at 90°C, followed by 30 cycles of 30 s at 94°C, 30 s at 60°C, 60 s at 72°C. The other commercial kits employ 3-step cycling and 30 cycles, with hold times ranging between 5 and 60 s (annealing) and 15–60 (extension), respectively.

The fast-cycling *Taq* mutants were found to be much less resistant to heparinized blood. The difference was more pronounced for single-site mutants (≤2% for heparinized blood vs. 15–45% EDTA-blood) than for combinatorial mutants (10–30% vs. 50–65% for heparin- vs. EDTA-blood, respectively). Presumably, heparinized blood poses a greater challenge due to the combined effects of these known inhibitors (blood and heparin; Satsangi et al., 1994). Resistance to heparinized blood decreases in the following order: 1C2, 2C2 (25–30%) > 42, 3B (10%) > G59W, E507K, F794I (2%) > V155I (≥1%) > wild-type *Taq* (<1%; Table 3), indicating that additional mutations (along with E507K) are essential to overcoming the inhibitory burden posed by heparinized blood. *Taq* 1C2 DNA polymerase has been incorporated into a commercially-available master mix (SureDirect PCR, Agilent Technologies), designed for amplification of genomic targets including cfDNA from large volumes of blood. When compared to other blood PCR kits, we found the majority can amplify the 232 bp Quantos target from 2.5 and 25% EDTA- and heparin-treated blood (Figure 6). Only the *Taq* 1C2-based formulation could amplify an endogenous target from 45% blood (heparin-treated).

## Discussion

In this report, we describe the properties of *Taq* DNA polymerase mutants evolved to self-replicate under abbreviated cycle times. Mutations emerging from CSR selection (G59W, V155I, L245M, L375V, E507K, K508R, E734G, F749I) were randomly combined and subjected to a final round of selection employing 15 s anneal-extension times. The fastest-cycling combinatorial mutants (*Taq* 42, 1C2, 2C2, and 3B) were shown to readily amplify genomic DNA targets up to 300 bp using abbreviated (≤10 s hold times) two-step cycling protocols on fast-ramping instruments (Figures 1, 2). When tested individually, the fast-cycling phenotype could be attributed solely to the E507K mutation (Figure 1B). Further characterization revealed that compared to wild-type, *Taq*E507K exhibits a dramatic (~90-fold) reduction in *K_d_* for primer-template (measured in the absence of nucleotides), and only moderate (~1.7-fold) or no improvement in polymerization rate or processivity, respectively. These findings suggest that *Taq*'s affinity for primed-template, rather than catalytic rate, is a limiting factor in PCR amplification under fast-cycling conditions.

A previous study concluded that E507 plays a role in primer-template binding, as substitution of E for Q improves *Taq's* RNA-dependent 5′ nuclease activity without altering DNA-dependent 5′ nuclease activity or RNA- and DNA-dependent DNA polymerase activities (Ma et al., 2000). This result would be expected if the amino acid side chain at position 507 comes in close proximity to the template strand, and substituting amine for negatively-charged oxygen reduces discrimination against the 2′OH of the ribose sugar. The crystal structure of *Taq* large fragment shows that E507 resides in the H1H2 loop of the thumb domain, which interacts with the distal portion of the primer-template duplex in both open and closed forms of binary and ternary complexes (Li et al., 1998). In this model, the peptide carbonyl of E507 comes in close proximity (3.8 Å) to the phosphate moiety between the 6th and 7th nucleotide from the 3′ end of the newly-extended primer (Figure 4B). As a whole, these data suggest that the E507K mutation stabilizes the *Taq*-DNA binary complex by forming additional contacts with the distal (away from the active site) portion of the primed-template. Higher KCl (95 mM instead of 50 mM) may be required with *Taq* E507K mutants to reduce binding affinity for mis-annealed primer-template during PCR annealing steps. The importance of E507 residue in protein/primer-template interactions has been shown in other DNA polymerase families as well. For example, in a study by Cozens et al. (2012), an analogs mutation to *Taq* E507K was made in the thumb domain of *Thermococcus gorgonarious* (family B DNA polymerase; E664K), which transforms *Tgo* into an RNA polymerase by lowering the *K_d_* for non-cognate RNA/DNA duplex and lowering the *K_m_* for ribonucleotide incorporation. This mutant was also capable of translesion synthesis across an abasic site or thymidine dimer.

As discussed above, CSR has been shown to exert strong selective pressure on polymerase fitness, enriching for variants that self-replicate with sufficient fidelity and catalytic efficiency to remain in the gene pool. For example, selected traits such as improved thermostability and increased tolerance to inhibitors have been shown to evolve with no cost to fidelity or catalytic efficiency (*K_cat_*/*K_m_*) (Ghadessy et al., 2001; Baar et al., 2011). CSR selection for tolerance to individual inhibitors has also been shown to produce broad spectrum resistance, presumably because survival requires self-replication in the presence of CSR emulsifiers, stabilizers, and bacterial debris (e.g., denatured protein, nucleic acid, and membrane lipid in the aqueous PCR compartments). In a striking example, CSR selection with bone powder produced a chimeric polymerase (2D9) with broad tolerance to a variety of environmental inhibitors, including humic acid, coprolite, peat extract, clay-rich soil, cave sediment, and tar, but surprisingly not to inhibitors in whole blood (Baar et al., 2011). Despite 81 amino acid changes, 2D9 exhibited comparable fidelity and processivity to wild-type *Taq*, consistent with the intrinsic requirement for polymerase fitness. A broad resistance spectrum implies a common mechanism of inhibition (for bone and soil extracts), prompting the authors to speculate that non-specific binding of inhibitors to protein and/or nucleic acid may sequester *Taq* or DNA template and prevent PCR amplification (Baar et al., 2011).

Encouraged by these reports and others, we assayed our CSR-selected mutants for inhibitor resistance. In addition to fast-cycling, the E507K mutation was shown to improve resistance to NaCl (tolerates up to 100 mM) and to inhibitors in whole blood (tolerates up to 45% (v/v) EDTA-blood). Individual mutations G59W, V155I, and F749I also confer blood resistance, but the magnitude of improvement is less (by 3-fold) than for E507K, and no corresponding increase in NaCl tolerance was observed (*Taq* L245M not tested). Heparinized blood posed a significant challenge due to the combined inhibitory effects of blood and heparin, as shown by the drastic difference in blood volumes tolerated by *Taq* E507K (22.5 μl EDTA-blood vs. 1 μl heparin-blood per 50 μl PCR). Apparently, increased DNA-binding affinity and NaCl-resistance conferred by the E507K substitution is insufficient to overcome the inhibitory burden, and additional mutations are required for amplification from larger (>5 μl) volumes of heparinized blood. Not all mutations or mutation combinations have been tested, but our results to date implicate G59W and 1–3 additional mutations (V155I, L245M, and/or F749I) in improving tolerance to heparinized blood. Compared to *Taq* E507K, *Taq* 3B (E507K *plus* V155I, L245M, F749I) can amplify genomic targets directly from 5% or 8% more EDTA- or heparin-blood, respectively. Adding the G59W mutation (*Taq* 3B *plus* G59W) further improves tolerance, allowing *Taq* 1C2 to amplify from 15% (EDTA-treated) or 28% (heparin-treated) more blood compared to *Taq* E507K.

Heparin and hemoglobin/hemin (the most potent inhibitors in blood) are thought to mimic and compete with duplex DNA for binding to the polymerase active site (Byrnes et al., 1975; Akane et al., 1994; Satsangi et al., 1994; Ghadessy et al., 2001). *Taq* mutants with increased tolerance toward heparin or blood have been described previously. After mutagenizing four amino acids implicated in cold-sensitivity and overall performance, Kermiekchiev et al. identified several substitutions at E708 that dramatically (>30-fold) improve resistance of *Taq* E626K/I707L and KlenTaq (N-truncated *Taq*) E626K/I707L to blood, hemoglobin/hemin, soil extract, and humic acid (Kermekchiev et al., 2009). Although no kinetic data were provided, results of a competitive filter-binding assay suggested that generic resistance to PCR inhibitors was related to increased affinity for DNA rather than diminished binding to hemin or humic acid (Kermekchiev et al., 2009). With the possible exception of humic acid, *Taq* E507K mutant exhibits a similarly-broad spectrum of resistance compared to wild-type (*Taq* E507K vs. *Taq* E626K/I707L/E708Q: >45- vs. ~100-fold for EDTA-blood; 0 vs. 2-fold for SYBR Green; 10- vs. 32-fold for SYBR in the presence of blood; 2- vs. 8-fold for humic acid) in addition to increased affinity for primer-template. E708 mutants have been commercialized by DNA Polymerase Technology, Inc. under the trade names OmniTaq (*Taq* E626K/I707L/E708Q) and OmniKlenTaq (KlenTaq E626K/I707L/E708K) (Zhang et al., 2010).

As discussed above, E507 resides in the primer-template binding site where mutations are expected to modulate DNA binding affinity. It's less clear how substitutions at or near E708 contribute to cold-sensitivity or increased DNA affinity/inhibitor resistance, as this region lies at the hinge-point of the fingers, away from the fingertip (which contacts incoming nucleotide and single-stranded template) and portions of the thumb and palm domains that interact with primer-template (Kermekchiev et al., 2009). *Taq* E507K and E708Q mutants are further distinguished by relative resistance to heparinized blood. *Taq* E507K tolerates up to 45% EDTA-blood compared to 2% heparin-blood, suggesting the combination of heparin and blood-associated (e.g., hemoglobin/hemin) inhibitors saturates a common (mutually-exclusive) DNA/heme/heparin binding site. Alternatively, E507K confers blood resistance by lowering sensitivity to NaCl, and mutations at other positions confer resistance to DNA mimics by reducing binding affinity for heme/heparin. In contrast to *Taq* E507K, E708 mutants (OmniTaq, OmniKlenTaq) exhibit similar tolerances to blood treated with EDTA, heparin, and citrate, consistent with a unique mechanism of inhibitor resistance (Zhang et al., 2010).

The heparin-binding site of *Taq* was precisely delineated by Ghadessy et al. (2001), who used CSR selection with heparin to identify a *Taq* mutant (*Taq* H15) with 130-fold higher resistance to heparin and comparable (to wild-type *Taq*) affinity for DNA in BIACore and template-dilution assays. Heparin resistance-conferring mutations cluster in the DNA-binding domain, and four of six residues mutated in *Taq* H15 make direct contacts with either the primer (K540, N583) or template (D578, M747) strand in open and closed forms of the binary/ternary complex (Ghadessy et al., 2001).

By selecting for increased speed rather than blood/heparin resistance, we and Kermekchiev et al. (2009) identified mutations that confer increased DNA binding affinity and tolerance for blood; curiously, the E507K mutant retained sensitivity to heparin, while E708 mutants appear equally tolerant to EDTA-blood and heparinized blood. In contrast, Ghadessy et al. (2001) identified 6 mutations in H15 that collectively diminish heparin binding without altering *K_d_* (DNA), even though heparin is a DNA mimic and the DNA-heparin binding sites are thought to overlap. These findings can be reconciled if one assumes that *Taq* makes additional contacts with primer-template outside the DNA/heparin binding site. In the Ghadessy study, one or more of the H15 mutations (K540, D578, N583, M747) may lower affinity for DNA and heparin through the same mechanism, while the remaining mutations restore *K_d_* (equal to wild-type *Taq*) through additional interactions with primer-template. Residing close to K540, N583 and the 3′ primer terminus, E507 is close to the DNA-heparin binding site, and introducing a positively-charged side chain (E→K) may increase binding affinity for both DNA and heparin (lower heparin tolerance). In contrast to E507, E708 lies farther from primer-template binding site, where mutations that increase DNA binding affinity won't necessarily enhance heparin binding or sensitivity.

Among the other blood-resistant mutations identified here, F749I is of particular interest due to its location relative to the template strand. Although pointing inward, F749 is flanked by amino acids that interact directly with heparin (M747) or the template nucleoside opposing the incoming nucleoside triphosphate (R746, M747, N750). F749 is also thought to stack against I707(Kermekchiev et al., 2003), which may provide a causal link to the cold-sensitive (KlenTaq 706–708 mutants) and inhibitor-resistant (*Taq*/KlenTaq 708 mutants) phenotypes identified by Kermekchiev et al. (2003, 2009).

The contribution of mutations located outside the DNA-binding pocket is more difficult to rationalize. In this study, individual mutations G59W and V155I confer increased (by >15-fold) resistance to EDTA-blood, while G59W improves amplification from heparinized blood (by 3.5-fold) when added to *Taq* V155I/L245M/E507K/F749I. G59 and V155 reside in the 5′ structure-specific nuclease domain responsible for excising Okazaki RNA during lagging strand synthesis (Li et al., 1998). The 5′ nuclease domain was implicated in an earlier study showing that N-truncated *Taq* (KlenTaq; residues 279–882) can amplify from 10- to 100-fold more blood (up to 5–10%) compared to full-length *Taq* (Kermekchiev et al., 2009). KlenTaq also exhibits higher thermostability and lower processivity compared to wild-type, suggesting that point mutations in the 5′ nuclease domain can modulate several properties of *Taq* that contribute to PCR performance (Barnes, 1992). Mutations that increase PCR fitness may alleviate the effects of other blood-associated inhibitors (immunoglobulin G, lactoferrin, proteases) that inhibit PCR by unknown means (Al-Soud and Radstrom, 1998, 2001; Al-Soud et al., 2000).

### Conflict of interest statement

The authors declare that the research was conducted in the absence of any commercial or financial relationships that could be construed as a potential conflict of interest.
